# Re-evaluating the access imperative in healthcare in the United States

**DOI:** 10.1057/s41271-025-00612-7

**Published:** 2026-01-03

**Authors:** Allen Chen

**Affiliations:** University of California, Irvine, Irvine, USA

**Keywords:** Access, Healthcare, Equity, Social determinants, Disparities

## Abstract

Access to healthcare, defined by the Institute of Medicine as "the timely use of personal health services to achieve the best health outcome” represents one of the most critical issues facing modern societies. However, barriers to access are increasingly being recognized across all populations especially as the boundaries between technology, medicine, business, public health, and policy become blurred. Given that resource and infrastructural constraints have been well established to influence access, organizations have the responsibility to continually evaluate this concept in the context of inclusivity and social determinants of health. Ultimately, improving access requires thoughtful engagement from a myriad of stakeholders with the goal of prioritizing timely, equitable, personalized, and high-quality care, while empowering patients to take charge of their own health. While a profound challenge, the journey toward bridging the many gaps is just beginning, and how society re-defines the access imperative in healthcare in an ever-evolving landscape represents one of the foremost issues of the future. Indeed, the implications for society are tremendous given that access is central to quality of care, profoundly impacts the patient experience, and influences health outcomes. The purpose of this review is to outline the core issues that contribute to access focusing on barriers, social determinants, quality of care, and potential interventions.

## Introduction

Healthcare is a universal human right that constitutes a foundational cornerstone for societies. According to the World Health Organization, “the enjoyment of the highest attainable standard of health is one of the fundamental rights of every human being without distinction of race, religion, political belief, economic or social condition [1].” In this sense, access to care—defined by the National Academy of Medicine as "the timely use of personal health services to achieve the best health outcome—” represents one of the most important issues facing modern communities [2]. Due to the sheer breadth of stakeholders that influence the healthcare marketplace— providers, insurance companies, government regulatory agencies, health systems, industry partners, pharmacies, among others— the coordination required to optimize access is extraordinarily complex. From a practical standpoint, access is central to quality of care, profoundly impacts the patient experience, and influences health outcomes. The purpose of this review is to outline the core issues that contribute to access focusing on barriers, quality of care, and potential interventions.

## Who?

Whether patients are facing a minor illness or a life-altering medical situation, the desire to be seen quickly is understandably natural. Indeed, consumers across all industries are increasingly accustomed to a “right now” society where goods and services can be provided at a moment’s notice. Patients navigating the healthcare system are no exception. Unfortunately, medical delivery is notoriously sluggish, and delays in care are common.

Data from the Agency for Healthcare Research and Quality continue to show that approximately 15% of adults nationwide cannot access medical services in a reasonably rapid fashion [3]. In fact, wait times today for new adult primary care appointments in large metropolitan markets average almost 30 days and climb to more than 100 days in select regions [4]. It is thus not surprising that nearly 30% of American adults report having no primary care provider, and as of 2022, almost 20% had not seen any doctor during the past year [5].

The reasons underlying these statistics deserve further evaluation. Could the access problems outlined be attributed solely to disparities in supply and demand? Or do other more systemic issues contribute to these lags? Regardless of the answers to these questions, what is increasingly evident is that patients are prioritizing access like never before. McKinsey and Company surveyed nearly 3,000 healthcare consumers on which criteria mattered to them when choosing a primary care provider. Out of 20 options, respondents consistently identified “appointment availability” and “appointment times that meet your needs” as among the top factors [4]. Additional data from Accenture demonstrated that nearly 2 in 3 consumers would switch healthcare providers for the ability to obtain an appointment quickly when needed [6]. Similarly, Kleij et al. conducted a systematic review of discrete choice experiments analyzing patient preferences to identify factors that could make health services delivery more responsive in the primary care setting [7]. Among the 18 studies, the authors found that the most commonly applied structure attribute was "waiting time” for appointment.

Yet as health systems struggle with cost containment and workforce shortages, it is prudent to better understand patient expectations for access. Unsurprisingly, studies have also suggested that a gap between perception and reality might exist with respect to how patients and providers value access [8–10]. Barry et al. showed that even when physicians think that they are providing timely service, this sentiment, in fact, might not be shared by patients [8]. In a survey study conducted at 2 community-based, ambulatory internal medicine clinics, the authors showed that patients expected to be seen sooner than physicians thought necessary for many common chronic medical conditions. They concluded that patients and physicians often have mismatched perceptions of the appropriate timing of office visits.

Numerous studies have attempted to define challenges in providing quality access. Hempel et al. convened an inclusive stakeholder panel to understand the dimensions and establish definitions of access management [11]. The investigators showed that the literature varies in access definitions, but the temporal measure “time to third next available appointment” was consistently used as an indicator. Oliver et al. similarly performed a cost-utility analysis to examine trade-offs that patients might consider during appointment bookings in 2 urban family medicine clinics [12]. Using 6 different simulated clinical scenarios across a number of key access and continuity attributes, they showed that patients preferred timely access over other attributes in the majority of cases. However, Gerard et al. showed that having the ability to see a specialist of choice and to maintain a relationship with a preferred provider were also influential to patients [13]. Notably, preferences varied by gender, employment, and career status, demonstrating that defining “access” is not quite as straight-forward as it seems.

Ultimately, a patient-led definition is essential in order to set objectives to improve access and to better understand potential barriers and inequities. Levesque et al. established a conceptual framework to outline the dimensions that relate to patients navigating the continuum of the healthcare environment [14]. The authors proposed a foundation integrating 5 dimensions of accessibility: approachability; acceptability; affordability, appropriateness, and availability and accommodation. These were superimposed on 5 corresponding factors that were patient-centric: ability to perceive; ability to seek; ability to reach; ability to pay; ability to engage. Using this model, the researchers suggested that the concept of access could be synthesized to improve care across populations.

## What?

**Fig. 1:**
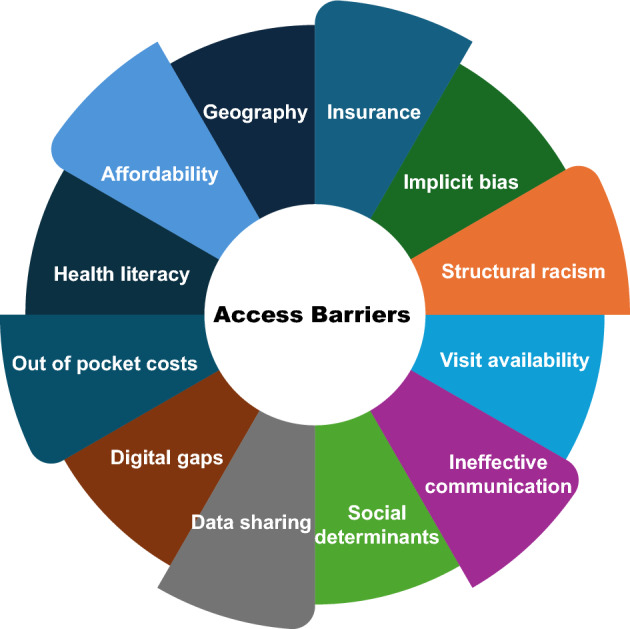
Barriers to access in healthcare

The barriers to access in healthcare are multi-faceted, broad, and commonly overlapping (Fig. 1). While commonly cited obstacles include those related to insurance coverage, affordability, social determinants, technical literacy, and/or provider availability, this list is by no means comprehensive. As importantly, more than one factor often contributes to delays in obtaining care depending on individual circumstances.

Although the passage of the Affordable Care Act (ACA) in 2010 led to improvements in coverage, a significant proportion of the public lack health insurance [15]. For others, just having insurance does not necessarily mean that access is guaranteed. For instance, studies have shown that prior authorization, which negatively impacts the ability of patients to see specialists, obtain diagnostic studies, acquire medications, and/or even see providers or start treatment can lead to major delays in care [16–18]. According to survey data of over 1000 practicing physicians from the American Medical Association, more than half of respondents reported that prior authorization has “impacted patient job performance [19].” With respect to access, a staggering 94% of physicians stated that “delays in care” resulted from prior authorization. As concerningly, more than three-quarter of the physicians reported that “treatment abandonment” occurred because of prior authorization; and more than one-third reported that prior authorization led to a serious adverse event including hospitalization, disability, and/or death for a patient while waiting for care.

The escalating cost of healthcare, particularly high out-of-pocket patient costs, is another well-documented access barrier [20]. One survey from West Health and Gallup found that 29% of adults, particularly those from lower income brackets, reported putting off medical treatment because of out-of-pocket costs between 2001 and 2021 [21]. Given data from the Commonwealth Fund showing that the United States has the starkest income-based health disparities compared to other similarly developed nations, the impact on access is profound [22]. Indeed, 46% and 27% of American adults regularly skip or have skipped a medical visit, test, treatment, follow-up, or prescription fill solely because of cost among low-income and high-income earners, respectively.

**Fig. 2:**
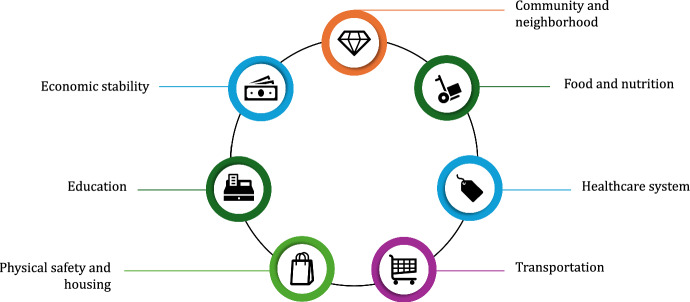
Social determinants of health

The influence of societal factors in creating inequities in access has also been well established [23]. Social determinants of health (Fig. 2) including factors related to income, education, employment, housing, and transportation, among others, have been shown to powerfully influence access and moreover are often embedded across generations [23–27]. Along these lines, the importance of geography cannot be understated as residents of lower socioeconomic status communities generally have poorer health outcomes than those living in more affluent neighborhoods. A person’s immediate surroundings also dictate lifestyle factors such as access to healthy foods, opportunities for physical activity, safe transportation, and other conditions such as water and air quality [28]. All of these variables can affect access to healthcare. Patients in rural areas have also cited transportation and work-related concerns as a key limit on the ability to access preventive care and treatment [29, 30]. Indeed, statistics from the American Hospital Association estimate that approximately 3.5 million patients go without care because they cannot access transportation to their providers [31].

Stigma and bias have been to influence access, including discrimination based on race, immigration status, sex, gender, and sexual orientation [32]. A position paper from the American College of Physicians (ACP) outlined how cultural factors hamper access to care and affect patients’ willingness and ability to seek specialized support such as mental healthcare services or pharmacologic therapy [33]. More specifically, the ACP underscored the importance of increased efforts to address urgent public health threats, including injuries and deaths from firearms; environmental hazards; climate change; maternal mortality; substance use disorders; and the health risks associated with nicotine, tobacco use, and electronic cigarettes.

Notably, nearly half of healthcare workers in the United States have witnessed racial discrimination against patients and say this is a crisis or major problem, according to the Commonwealth Fund [34]. According to findings from another large, nationally represented survey published by the Kaiser Family Foundation in 2023, approximately 60% of Black adults, half of Native American and Latino adults, and 40% of Asian adults admitted to preparing for possible insults from providers or staff and/or felt they must be careful about their appearance to be treated fairly during healthcare visits [35]. Furthermore, the survey found that patients who experienced discrimination were more likely to have reported feelings of anxiety, loneliness, and depression.

Structural factors also can contribute to barriers to access. For instance, historical policies related to redlining (to designate “desirable” and “undesirable” neighborhoods) have led to both racial segregation and disparities in access to resources and services, like high-quality hospitals [36]. The implications on healthcare access have been shown to be profound [36–38]. As one example, the Primary Care Development Corporation demonstrated that the rate of uninsurance—which can directly impact access to healthcare and healthcare affordability—was much higher in formerly redlined districts, with 18% of adults in those areas saying they lack payer coverage [39].

As importantly, implicit bias can also instill distrust in medicine and dissuade patients of color from accessing care [40]. In these cases, patients who feel that the healthcare system only caters to the privileged might fall through the cracks due to a perceived indifference for their beliefs and unique backgrounds. For instance, medical appointments can routinely conjure up emotions of fear and despair that can be exacerbated in certain underserved communities even accounting for intergenerational trauma [41]. Given that 1 in 5 households in the United States speaks a language other than English at home, the influence of cultural competency in creating access problems must be acknowledged [42].

Indeed, an often-underappreciated aspect of access to health services pertains to provider communication. Even outside of a face-to-face encounter, the provider–patient relationship requires continued nurturing. With the advent of digital tools to enhance access, patient expectations for timely communication continue to expand. For instance, questions such as How and when will test results be delivered? How does the physician handle routine and urgent questions? Does the physician communicate by e-mail correspondence? all have implications regarding access. Studies have consistently shown that delayed communication contributes to confusion and depersonalization for patients [43–45].

Even when patients have the means and motivation to seek out medical care, a lack of available and/or convenient appointments can hinder access. While most policy makers point to a healthcare provider shortage and a workforce that is unable to keep up with the demands of a growing and aging population as the primary cause, other factors contribute as well. For instance, the use of inefficient scheduling systems predicated on obsolete templates and processes not optimizing analytics must also be considered. These gaps are particularly problematic for patients residing in “healthcare deserts” such as rural areas.

Finally, issues related to information flow can contribute to access barriers for patients, particularly those with more complex problems [46–48]. Given that patient visits are episodic in nature and require the sharing of information between providers, the lack of a centralized medical record system can readily contribute to poor information exchange between providers. For instance, problems with interoperability and integration with medical record storage systems can lead to increased fragmentation of visits. The lack of compatibility between electronic health records and hospital management software can result in significant impediments in accessing patient information. These inaccuracies can lead to miscommunications with patients and payers, as well as delays in providing care, duplicative testing, and/or missed opportunities for reimbursement.

The lack of technical literacy for many patients, particularly those on the lower end of the socioeconomic spectrum, can hinder activities such as scheduling appointments, checking results, and/or communicating with providers—tasks that are increasingly digitized in modern healthcare [49]. Indeed, underrepresented minorities have been shown to have more difficulty accessing their medical records online [50]. More recently, the term “digital redlining” was introduced to describe racialized inequities in access to technology infrastructure, including access to healthcare, education, employment, and social services [51].

## Why?

Studies have consistently shown that improved access leads to higher quality care, improved health outcomes, and reduced costs [52–54]. From a consumer perspective, the ability to be seen quickly and attentively represents the pinnacle of patient-centric care; and the positive impact on patient satisfaction has been well established. Conversely, patients who are subjected to excessive delays are more likely to become frustrated, anxious, or depressed. They are also more likely to file complaints or possibly even seek legal recourse [55].

The timely receipt of care has been shown to correlate with improvements with regard to a variety of different endpoints over many conditions [56–58]. Numerous studies, for instance, have shown that delays in cancer treatment are detrimental across nearly every disease site [58–62]. A published analysis that reviewed 34 different studies including over 1 million patients showed that every 4 weeks delay in treatment can increase the risk of death by 10% with that number incrementally rising with continued lags [62].

The most conclusive data reporting on the impact of delays in access on patient care stem from the Veterans Health (VA) administration [63–66]. According to one study on geriatric veterans, patients aged 70 to 74 were nearly 10 percent more likely to have a stroke when visiting facilities with longer waiting times [63]. An association between an increased risk of acute myocardial infarction and wait times was also established. Furthermore, at approximately 30 days of appointment wait time, older patients experienced higher mortality rates and hospitalization events. Studies from the VA have also shown that diabetes can be prevented or managed better with timely care [64]. Penn et al. showed that as wait times improved in recent years, there has been a parallel improvement in patient satisfaction validating the premise that patients value access [65]. Conversely, Wong et al. showed that patients reporting longer usual wait times were significantly more likely to seek care elsewhere [66].

Some of the most striking literature on how delays in care affect outcomes arose from the COVID-19 pandemic [67–71]. Numerous studies have since shown increased mortality rates from non-COVID causes as a result of neglected and/or delayed care [71–73]. It was also shown that preventive services such as screenings were underutilized during the pandemic [74–76]. The implications with respect to cancer prevention, cardiovascular health, and weight management, among others are obvious.

It must be recognized that delays in care often lead to higher and/or avoidable healthcare costs, both for patients and the system. Additionally, those who are experiencing excessive delays for appointments are more likely to leave the health system for care and/or to skip their previously scheduled encounters, leading to operational efficiencies which could prove costly over time [66]. One study found that patient ‘no shows’ cost the United States healthcare system more than $150 billion a year and individual physicians an average of $200 per unused time slot [77]. Finally, studies have shown that a considerable proportion of visits to the emergency room, a cost- and resource-intensive use of services, could have been avoided with more routine use of primary care for low-acuity needs [78]. The perception held among many Americans that it is easier and more convenient to obtain care in the emergency room than in the outpatient setting is pervasive and leads to waste [79–81].

Within general healthcare, patients waiting for diagnosis, surgery or treatment experience increases in negative affect, including anger, frustration, fear, stress, anxiety and depression, as well as reduced self-esteem, which worsened the longer they waited [82]. An increasing amount of literature has also emerged specifically demonstrating the detrimental consequences for adults awaiting support for their mental health [83–85]. Additionally, longer wait times can lead to feelings of disillusionment with the healthcare system causing patients to skip out entirely on such encounters as routine check-ups and screenings which can potentially result in serious long-term health consequences. In this sense, improved access strongly promotes patient engagement, a critical element that prioritize public health across society.

## How?

Technological advances have the potential to improve access to quality care while prioritizing patient well-being, and enabling providers to make more informed, evidence-based decisions. Digital communication tools, such as mobile health apps, telemedicine, and online health information resources, have gained significant popularity and are increasingly being integrated into healthcare delivery systems. By harnessing the power of technology, digital communication tools have the potential to enhance health literacy, improve the patient-provider relationship, and ultimately lead to better health outcomes. The WHO recently published a framework of “e-Health” for improved health service delivery, describing the potentially powerful contributions of digital platforms to each of the health system attributes (service quality, efficiency, equity, accountability, sustainability, and resilience) at different levels—the individual, the service provider, the health-care organization, and the overall health system [86].

Optimizing means of asynchronous patient communication and monitoring through technological solutions should also be a priority. Given the increasing backlog of appointments faced by many health systems, resorting to these methods can fill a vast need. Patient portals and secure messaging platforms allow patients to communicate directly with their providers securely and readily at their respective convenience. They can be used to enhance trust and to foster a stronger patient–provider relationship without the need for formal office visits [87, 88].

Innovative open access scheduling systems utilizing online self-service applications have also been proposed as a means to streamline the appointment process—so patients can schedule, reschedule, or cancel appointments whenever it is convenient for them, which is often outside provider office hours [89–91]. By outlining available appointments in a visible and transparent fashion, these systems empower patients to make user-friendly decisions. These have the added benefit to optimize unused capacity, particularly as machine learning and artificial intelligence (AI) is integrated into pathways. Self-service tools can also reduce administrative overhead, so staff can focus on critical tasks that require a human touch. Similarly, digital pre-registration allows patients to complete paperwork from home, where they have access to their medical records and insurance information. AI-based tools can increasingly pre-fill much of these data, saving time and preventing errors. Indeed, the potential of AI to register, triage, and even diagnose patients in the clinical setting is just starting to be explored. The popularity of same day access—which allows patients to be seen within hours from scheduling an appointment—has also been increasingly demonstrated [92, 93].

The importance of expanding provider capacity—through training of more qualified physicians, relying on non-physician extenders such as nurse practitioners, physician assistants, and patient navigators, and optimizing scheduling templates to match supply and demand—is unquestioned [94]. At the most basic level, the ability to satisfy the needs of an expanding patient population depends critically on alleviating any workforce shortage. The need to address provider burnout is also essential to minimize turnover and to maintain staffing. Without purposeful efforts to prioritize provider recruitment and as importantly, retention, the ability to address access will naturally be limited.

To further ameliorate the logistical barriers limiting access, social programs such as housing services and ride share options should actively be implemented. Patients who are physically unable to drive, who face financial barriers, or who otherwise cannot obtain transportation to the provider office often go without care. Through collaboration with non-emergency medical transportation providers and emerging rideshare companies like Uber and Lyft, healthcare providers and payers have the ability to design programs to bridge the access gap. One study evaluated the impact of rideshare-based medical transportation on the proportion of Medicaid patients attending scheduled primary care appointments and showed significantly improved rates of compliance [95]. Partnership opportunities with payers, community organizations, local nursing homes, federally qualified health centers, among other institutions should also be explored to align interests with respect to improving access.

Initiatives to improve data usability by centralizing information into integrated servers can make the healthcare system more efficient, thus optimizing downstream services such as patient scheduling. Given the vast amount of patient-related data associated with encounters, the establishment of a nationalized database of health records could also revolutionize access, allowing the sharing of information among providers instantaneously.

Moreover, efforts to thoughtfully promote cost and price transparency are needed to make the healthcare system more open and friendly for all. While patient demand for healthcare services generally does not respond in the same manner as consumer demand for other goods in terms of price elasticity, price transparency, in theory, will enable patients to shop for the most effective, lowest-cost healthcare available and drive expenses down as providers compete for market share. The implications with access are immense. After all, when patients are faced with not knowing what they will owe for their care until they receive a bill weeks later, encounters are frequently delayed or skipped altogether. It is thus no surprise that price transparency is supported by over 90% of Americans [96]. While the ACA required individual hospitals to make prices transparent by publishing their “chargemasters,” or list prices, for all the services they provide, the resultant effect has arguably increased confusion. This is because the unwieldy labyrinth of information published, listing thousands of goods and services posted on thousands of websites is of little practical benefit for patients, who are more interested in out-of-pocket costs. What is needed instead are efforts to provide patients an accurate, personalized breakdown of their estimated financial responsibility prior to being seen. This should be accompanied by initiatives to increase patient engagement, provide real-time assistance with interpreting both outcomes and cost information, compare available treatment and provider alternatives, and couple price information with quality metrics of specific relevance to enable making fully informed decisions. Provider organizations can also leverage these data to address inequities on a community and population-based level. Only through standardization of these efforts will consumer empowerment truly be optimized.

Along these lines, it is critical that any discussion on access improvement include a review of the financial considerations impacting care. On one end of the spectrum are patients who tolerate excessive delays due to an inability to cover costs and/or weather disruptions in their employment; conversely, the emergence and rapid growth of concierge medicine for individuals with substantial discretionary resources is a direct manifestation of just how meaningfully some segments of society value access—and personalized service. Indeed, given that concierge medicine is typically only available to the privileged (who can pay for services in cash), it is clearly not the answer to the broader access problem. For the larger population, the role of insurance in impeding or facilitating access is more complex. In general, individuals who are fortunate enough to have comprehensive, low or no deductible insurance usually have fewer access issues than individuals who have coverage with high deductibles. Likewise, individuals with high deductible coverage insurance products, have fewer barriers to access than the uninsured. Therefore, the promotion of access across the population will require a discourse on expanding high-quality insurance coverage to more individuals and to strategically devise solutions for excessive out-of-pocket costs. Whether a national mandate will solve these issues is the source of ongoing debate among both scholars and policy makers [97].

It is likely that current practices of provider compensation also have repercussions on access. For example, the revenue value unit (RVU) system of physician payment generally rewards providers for throughput and productivity. However, the effects on access may actually be counterintuitive as the possibility of overloading the system and/or even promoting low-value or unnecessary care must be recognized. In this regard, the risk of catering to unrealistic patient expectations, leading to an environment in which care is rendered regardless of whether it is appropriate or justified, could be amplified in these RVU-based models of compensation. Thus, the ability to decrease or eliminate low-value and/or unnecessary visits is critical to expand access, especially when the provider workforce remains unchanged in number. Ultimately, a system of reimbursement that truly rewards quality, efficiency, and patient satisfaction might lead to less patient visit but result in more effective and appropriate healthcare—thereby optimizing capacity with the goal of logically improving access.

Lastly there are a growing number of initiatives to address social determinants to promote health equity. Models under the Center for Medicare and Medicaid delivery system are increasingly addressing social needs and implementing community-based preventive programs [98]. Recently, numerous states required Medicaid managed care plans to screen for and/or provide referrals for social needs, and a recent survey found that nearly all responding plans reported activities to address social determinants of health [99]. Government initiatives to address the inequities in rural and inner-city regions of the United States also have the potential to improve access for these critical populations [100–102]. With regard to access, educational initiatives are being explored to help patients understand the options for care delivery and the varying caliber of services available at different care facilities [103, 104].

## Conclusion

Forecasting the future of healthcare is an imperfect science. As the boundaries between technology, medicine, business, public health, and policy become increasingly blurred, society has the responsibility to critically analyze priorities in a complex and fast-changing environment, while maintaining focus on the human element of the patient-provider experience. For the patient, this begins with access, which is increasingly recognized as a cornerstone of high reliability healthcare. In short, improving access is about determining how to best empower patients to take charge of their own healthcare. This requires thoughtful engagement from a myriad of stakeholders with the goal of ultimately prioritizing timely, equitable, and high-quality care. While a profound challenge, the journey toward bridging the many gaps is just beginning. As demonstrated in this review, the utility of access as a catalyst to drive toward a quality, efficient, and equitable patient-centered delivery system will require significant healthcare reform at multiple levels of society. Indeed, how populations re-define the access imperative in healthcare in an ever-evolving landscape represents one of the foremost issues of the future.

## Data Availability

No datasets were generated or analyzed during the current study.
